# Hemorrhage, Fistulization, and Rupture: Management of a Cascade of Complications from a Single Pancreatic Pseudocyst

**DOI:** 10.7759/cureus.109429

**Published:** 2026-05-22

**Authors:** Deepti Sudhakar, Jay Desai, John Culhane

**Affiliations:** 1 General Surgery, Saint Louis University School of Medicine, Saint Louis, USA; 2 General Surgery, Sisters of Saint Mary (SSM) Health Saint Louis University, Saint Louis, USA

**Keywords:** acute surgical abdomen, hemorrhagic pancreatic pseudocyst, intraperitoneal rupture, pancreatic pseudocyst (ppc), pancreatic pseudocyst’s fistula

## Abstract

Although individual complications of pancreatic pseudocysts are well-documented, the occurrence of multiple complications from a single pseudocyst is exceptionally rare. This report illustrates the surgical management of a cascade of life-threatening complications arising from a single pancreatic pseudocyst of gastric fistulization, intraperitoneal rupture, and splenic involvement following intracystic hemorrhage. A 42-year-old female patient with chronic alcoholic pancreatitis and a known pancreatic tail pseudocyst presented with severe abdominal pain and emesis. The workup revealed active extravasation from a splenic artery branch that was managed with arterial embolization. The patient returned three days following embolization with sepsis and peritonitis. An emergent exploratory laparotomy revealed an infected hematoma and intraperitoneal rupture of the pseudocyst that had fistulized into the stomach with splenic involvement, requiring a distal pancreatectomy, splenectomy, and wedge gastrectomy. Postoperatively, the patient’s nasogastric tube (NG) perforated the suture line, necessitating further repair, but she otherwise recovered uneventfully. Arterial embolization can achieve hemostasis in cases of hemorrhagic pseudocysts, but patients remain vulnerable to further pseudocyst complications. Definitive management involves drainage or resection. The management of ruptured pseudocysts should be determined based on patient stability and involvement of surrounding structures.

## Introduction

Pancreatic pseudocysts are a well-known sequela of acute and chronic pancreatitis. They form when inflammation or obstruction of pancreatic ducts leads to extravasation of pancreatic fluid into well-defined collections surrounded by a non-epithelialized inflammatory wall that typically develops after four weeks after an episode of pancreatitis [1]. While most pancreatic pseudocysts spontaneously resolve, complications may arise, including infection, rupture, hemorrhage, fistulization, and mass effect on surrounding structures [2].

Pancreatic enzymes from a pseudocyst and pressure necrosis may weaken the adjacent arterial walls and form a pseudoaneurysm that is at an increased risk of rupturing into the cyst, forming a hemorrhagic pancreatic pseudocyst, or into other structures such as the peritoneum. The splenic artery is the most frequently involved blood vessel, followed by the gastroduodenal artery and pancreaticoduodenal arteries [3]. The first-line treatment for hemodynamically stable patients is arterial embolization, which is a minimally invasive, image-guided procedure in which angiography is used to first identify the bleeding vessel and then embolic agents such as metal coils or medical glues are delivered via catheter to effectively seal off the bleeding vessel and achieve hemostasis.

Fistulization occurs when enzymatic digestion forms an abnormal communication between the pseudocyst and adjacent hollow viscera. Rupture involves the loss of integrity of the pseudocyst wall and subsequent spillage of its contents. Both are extremely rare and life-threatening complications that occur in less than 3% of pancreatic pseudocysts and carry high morbidity due to the risk of secondary complications such as infection, sepsis, and ascites based on where specifically the pseudocyst empties into [4].

We present the rare case of a 42-year-old female patient with chronic alcoholic pancreatitis whose initial spontaneous hemorrhagic pancreatic pseudocyst was followed by a rapid cascade of fistulization, rupture, and splenic hilar involvement. This unique case discusses the management of rapid-onset pseudocyst complications in the acute care setting and highlights surgical strategies for handling highly friable, inflamed tissue amidst enzymatic degradation.

## Case presentation

A 42 year-old Caucasian female patient with chronic alcohol-related pancreatitis and a known tail pseudocyst presented to the emergency department with abdominal and back pain associated with non-bloody emesis. Her symptoms began 14 hours prior to presentation as localized right lower quadrant pain that progressively worsened to a 10/10 in severity and spread diffusely throughout the abdomen with radiation to the back. She reported nausea with one episode of non-bloody emesis at home and a second upon arrival to the hospital. Her medical history was notable for metabolic dysfunction-associated steatotic liver disease, a prior splenic vein thrombosis (previously treated with a temporary course of anticoagulation, discontinued one year prior to presentation), a history of alcohol use disorder with five months of sobriety, and an active 10 pack-year smoking history. Family history was non-contributory, with no known hereditary predispositions to pancreatitis, malignancy, or hypercoagulable disorders. Surgical history included laparoscopic cholecystectomy.

On arrival, the patient was tachycardic to 109 bpm and hypertensive to 160/92 mmHg. Upon examination, she was uncomfortable and her abdomen was diffusely tender to palpation, most prominent on the right, without rebound or guarding. Laboratory evaluation was notable for lipase of 38 U/L (reference: 8-78 U/L), a white blood cell count of 8.6x10^9^/L (reference: 4-10.7x10^9^/L), and initially stable hemoglobin of 12.0 g/dL (reference range: 11.9-15.8 g/dL).

Computed tomography (CT) of the abdomen and pelvis with intravenous contrast performed 6.5 hours after presentation showed an 11x8 cm heterogeneous fluid collection abutting the tail of the pancreas and the posterior wall of the stomach with active extravasation, consistent with a hemorrhagic pancreatic pseudocyst (Figure 1). While initially stable for a planned visceral angiogram with arterial embolization, the patient acutely decompensated 7.5 hours after arrival with a drop in her systolic blood pressure from 160 to 100 mmHg and hemoglobin from 12.0 to 9.6 g/dL (reference: 11.9-15.8 g/dL).

**Figure 1:**
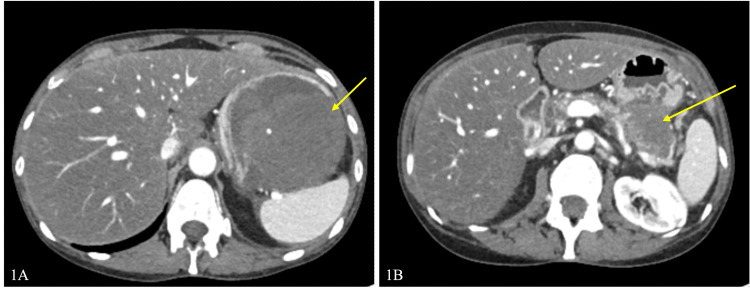
Hemorrhagic Pancreatic Pseudocyst on Contrast-Enhanced CT of the Abdomen (A) Axial contrast-enhanced CT image demonstrating a large heterogeneously attenuating fluid collection (arrow) with mass effect on the spleen. (B) More caudal slice showing the same collection (arrow) in close proximity to the tail of the pancreas and the posterior aspect of the stomach.

The patient was urgently taken to the interventional radiology suite where a visceral angiogram was performed, demonstrating active extravasation from a branch of the splenic artery (Figure 2). After unsuccessful attempts to select the specific distal hemorrhagic branch, the interventional radiology team performed proximal embolization of the splenic artery with Gelfoam and achieved hemostasis. The patient was then admitted to the intensive care unit where her vital signs and hemoglobin remained stable off pressors. She was discharged home on post-procedure day two with plans for outpatient follow-up and repeat imaging in one to two weeks.

**Figure 2:**
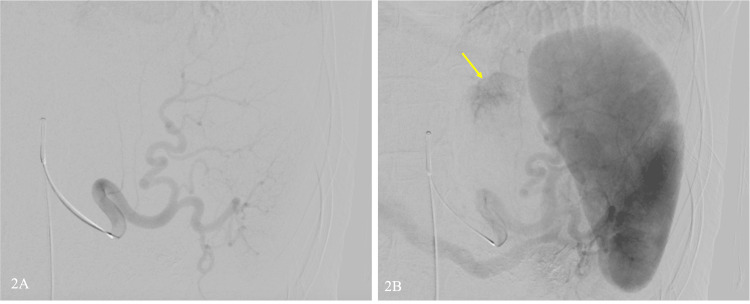
Splenic Artery Angiography (A) Early arterial phase of a selective splenic artery angiogram showing the splenic artery trunk and branches. (B) Delayed phase from the same study demonstrating active extravasation of contrast (arrow) arising from a superiorly coursing branch of the mid-portion of the splenic artery.

However, the patient returned to the emergency department one day after discharge with a sudden onset of severe abdominal pain, nausea, and non-bloody emesis. On examination, she was diaphoretic, with a rigid, peritonitic abdomen, severely tachycardic (heart rate of 150 bpm), and borderline hypotensive (systolic blood pressure of 100-105 mmHg). Laboratory evaluation was notable for leukocytosis of 16.7x10^9^/L (reference: 4-10.7x10^9^/L), hemoglobin of 9.4 g/dL (reference: 11.9-15.8 g/dL), and lipase of 15 U/L (reference: 8-78 U/L). CT angiography showed pneumoperitoneum and a hematoma with an internal focus of gas communicating with the gastric lumen that was concerning for a new fistulous tract from the pseudocyst to the stomach and gastric perforation (Figure 3). Based on these findings, the acute care surgery service performed an emergent exploratory laparotomy.

**Figure 3:**
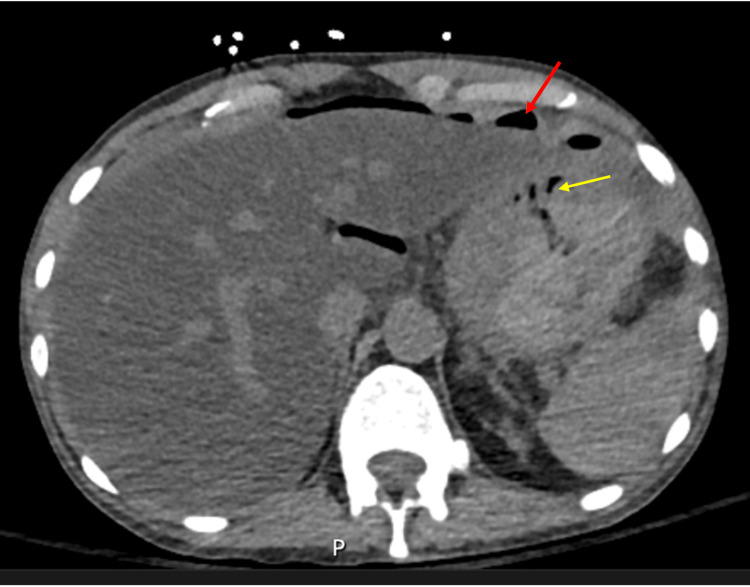
Pneumoperitoneum and Intracystic Gas on Contrast-Enhanced CT of the Abdomen Axial contrast-enhanced CT image demonstrating free intraperitoneal air (red arrow) and mottled gas (yellow arrow) within the heterogeneously attenuating fluid collection, suggesting a fistulous communication with the gastrointestinal tract.

A generous upper midline incision was made and upon entering the peritoneal cavity, approximately 500 mL of dark, murky fluid and 200 mL of what appeared to be an infected hematoma were evacuated and sent for culture. Surgeons also visualized dense adhesion of the pseudocyst to the greater curvature of the stomach and spleen. Further exploration of the pseudocyst revealed a peritoneal perforation, which communicated with the gastric lumen (Figure 4). The distal pancreatic tail was resected to excise what was most likely the source of the pseudocyst and the pancreatic remnant was oversewn with 2-0 Prolene (Ethicon, Inc., Raritan, NJ). A splenectomy exposed an approximately 15 cm anterior gastric wall perforation and the expulsion of gastric contents. Given the large perforation and the edematous, friable quality of the marginal tissue, a wedge resection of the greater curvature of the stomach was performed. To achieve a more secure and tension-free closure amidst significant enzymatic degradation, large bites were taken, and a single layer of #1 Vicryl sutures (Ethicon, Inc., Raritan, NJ) was placed in an interrupted fashion spaced further apart to avoid ischemia. The procedure concluded with an abdominal washout and placement of left upper quadrant and lesser sac drains, a large-bore nasogastric (NG) tube, and a distal Dobhoff feeding tube (Avanos Medical, Inc., Alpharetta, GA). Intraoperatively, the total estimated blood loss was 400 mL, which included 200 mL of acute hemorrhage and 200 mL of evacuated hematoma.

**Figure 4:**
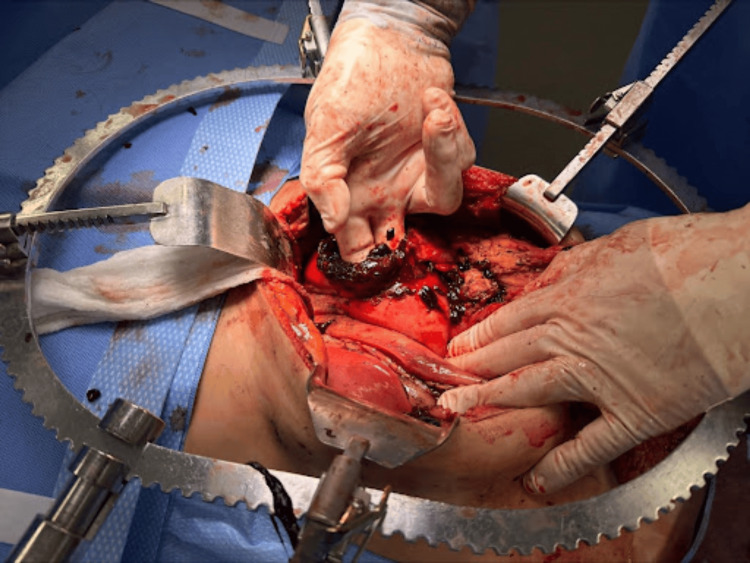
Intraoperative Findings During Exploratory Laparotomy Intraoperative view during exploratory laparotomy. The finger tips are within the large pseudocyst cavity, which has a visible communication with the posterior gastric lumen. Dark, hemorrhagic clot and necrotic material are visible, consistent with an infected, ruptured hemorrhagic pseudocyst.

Postoperatively, the patient was hemodynamically stable and gastric decompression was maintained with an NG tube while feeds and medications were administered through a post-pyloric Dobhoff tube. On post-operative day two, she developed worsening leukocytosis and left upper quadrant pain worsened by NG tube flushing. Bilious drainage from both of her Jackson-Pratt (JP) drains prompted a CT scan that revealed pneumoperitoneum and the nasogastric tube passing visibly through the repaired gastric perforation (Figure 5). She was taken back to the operating room emergently for an exploratory laparotomy. The peritoneal cavity was re-entered and the surgeons immediately encountered light gastric fluid with visible perforation of the NG tube through the center of the previous gastrotomy closure. Given that the gastrotomy edges appeared healthy, the NG tube was repositioned and the gastrotomy was closed with three #1 Vicryl sutures. A leak test was negative and intraoperative blood loss was minimal (10 mL).

**Figure 5:**
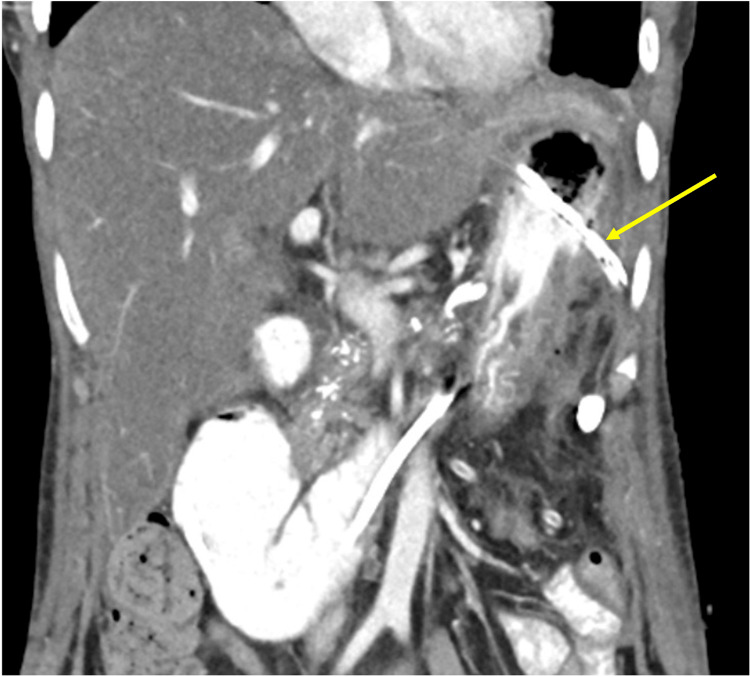
Pneumoperitoneum and Visceral Perforation on Contrast- Enhanced CT of the Abdomen Coronal contrast-enhanced CT image demonstrating the nasogastric tube traversing the gastric wall (arrow) into the peritoneal cavity and localized pneumoperitoneum.

The patient’s postoperative course was managed with a multimodal pain regimen. Intraoperative cultures from the evacuated hematoma grew light *Viridans streptococcus* and rare *Candida tropicalis*, organisms typical of upper gastrointestinal flora. This supports a mechanical rather than a primary gas-forming bacterial etiology for the intracystic air identified on preoperative imaging (Figure 3). The patient was treated with a four-day course of piperacillin/tazobactam, which led to the resolution of her leukocytosis. Antifungal therapy was briefly resorted to due to the proximal nature of the perforation, but a nine-day course of fluconazole was completed once formal cultures confirmed fungal growth. An upper gastrointestinal study on postoperative day five demonstrated trace extravasation at the lateral margin of the gastric fundus. Given the minimal volume of extravasation and the patient's lack of clinical symptoms, enteral nutrition was initiated through the Dobhoff tube and her diet was gradually advanced to oral intake with subsequent removal of the Dobhoff. The Jackson-Pratt (JP) drains, which had been bilious prior to the second ex lap, transitioned to low-volume serosanguineous output. She was discharged home on postoperative day 11 after receiving the standard post-splenectomy vaccine series. At her follow-up clinic visit, she was healing well and all surgical staples and drains were removed with no need for further follow-up.

## Discussion

Most pancreatic pseudocysts less than 4 cm in size spontaneously regress, so observation is recommended for stable, asymptomatic cases [5,6]. Intervention is required when pseudocysts become symptomatic or complicated by obstruction, hemorrhage, infection, or rupture. Minimally invasive drainage (endoscopic, percutaneous, or laparoscopic) is preferred for uncomplicated cases, while open surgical intervention is warranted for complicated pseudocysts [2].

The patient presented in this report suffered several complications of her previously stable pancreatic pseudocyst. Initial hemorrhage from a splenic artery branch was controlled with arterial embolization, which is the first-line treatment for hemodynamically stable patients. Hemorrhage is a rare complication that affects roughly 10% of pseudocysts and typically occurs when proteolytic enzymes released during pancreatitis erode and weaken the adjacent vasculature or, less commonly, when the pseudocyst wall itself directly invades into surrounding structures [3]. Hemorrhage carries high morbidity due to potential bleeding into the gastrointestinal (GI) tract, pancreatic ducts, peritoneum, retroperitoneum, or the pseudocyst itself [2]. Although embolization effectively achieves hemostasis in over 95% of cases, it does not resolve the underlying pseudocyst, leaving patients at risk for infection, rupture, mass effect, and an approximately 15% chance of rebleeding [7]. 

The patient’s early discharge following arterial embolization warrants critique, particularly given the high-risk nature of the remaining collection. The Revised Atlanta Classification moved away from a size-based threshold for drainage, and instead supports that intervention is indicated for pseudocysts that are symptomatic, complicated, or have impending complications [1]. Despite achieving hemostasis after embolization, this patient’s collection remained complicated by its large size, its composition of blood and pancreatic enzymes, and its significant mass effect on the adjacent gastric wall. These factors likely facilitated pressure necrosis and enzymatic degradation that led to the subsequent gastric fistulization and intraperitoneal rupture. Given the splenic artery’s contribution to gastric blood supply via the short gastric and left gastroepiploic arteries, embolization may have also contributed to devascularization and weakening of the gastric tissues, predisposing it to fistulization. Thus, arterial embolization should have served as a bridge to definitive drainage or resection rather than the endpoint of management.

Rupture occurs in less than 3% of pseudocysts and carries high morbidity, as it can precipitate peritonitis, sepsis, and multi-organ involvement [4]. Isolated rupture into the stomach often allows internal drainage and may be managed conservatively, but surgical or endoscopic intervention is required if the drainage is incomplete or if complications like infection occur. Similarly, isolated rupture into the retroperitoneum or peritoneum may be self-limited if there is no active leakage from the pancreatic duct. However, this patient’s situation was especially rare due to simultaneous splenic involvement. Splenic involvement can occur by direct pancreatic enzymatic erosion through the splenic capsule, vascular dissection, intrasplenic pseudocyst formation, or splenic infarction with liquefaction [8]. Surgical approaches with splenic involvement typically involve either distal pancreatectomy and splenectomy or external drainage and splenectomy. Percutaneous drainage offers a nonoperative approach, but has high rates of reintervention since it does not address the continued communication of pancreatic ducts with pseudocysts [8]. Distal pancreatectomy is preferred in cases of pancreatic tail necrosis, ductal disruption, non-communicating pseudocysts, or concern for malignancy. Drainage with splenectomy is typically preferred when the pancreatic tail remains viable and there is no ductal disruption. In this case, distal pancreatectomy was performed to eliminate the source of the pseudocyst, and the spleen was sacrificed to better visualize and repair the gastric defect.

This patient additionally suffered a postoperative complication of NG tube perforation of the gastric suture line. While the NG tube was placed intraoperatively such that it passed the area of rupture, manipulation during routine postoperative care may have contributed to the perforation. Techniques to ensure fixed positioning such as suturing the NG tube to the nasal septum or using a nasal bridle could have been implemented to prevent erroneous malpositioning. While wider suture spacing was chosen during the primary repair to preserve tissue perfusion, this likely increased the risk of perforation. This demonstrates the importance of balancing tissue perfusion with the soundness of repair when determining how much of the stomach to resect and when determining the spacing of sutures when handling friable edematous tissue.

A search of reported cases revealed only one previously reported case describing concurrent gastric perforation and splenic involvement requiring distal pancreatectomy, splenectomy, and total gastrectomy [9]. However, no published reports describing the specific combination of severe complications arising from a single pancreatic pseudocyst as observed in this patient were found. This case demonstrates the importance of early recognition of pseudocyst complications and the need for individualized management based on anatomical involvement and patient stability.

## Conclusions

This case underscores that while arterial embolization is effective for achieving hemostasis in a hemorrhagic pancreatic pseudocyst, it does not address the remaining hematoma. Patients remain vulnerable to secondary complications such as pressure-induced necrosis, rupture, and fistulization. Therefore, intervention with surgical or percutaneous drainage should be considered following embolization and close vigilance is necessary to anticipate further complications.

The rapid progression from a chronic stable pseudocyst to multi-organ involvement in this case highlights the volatile nature of enzymatic degradation in the acute care setting. While management of pancreatic pseudocysts typically involves drainage or resection, an individualized approach is required to manage more complex cases. Distal pancreatectomy and splenectomy may even be necessary for definitive removal of the source of complication. Early recognition and aggressive surgical intervention are key for improving outcomes in such rare complications of pancreatic pseudocysts.
